# Crystal structure determination of an Fe^II^ azo aldehyde complex [Fe(C_14_H_11_N_2_O_3_)_2_(H_2_O)_2_] by MicroED

**DOI:** 10.1107/S2056989026004755

**Published:** 2026-05-12

**Authors:** Kazuki Ishikawa, Fumishi Yoshizawa, Reon Aihara, Norihito Funato, Daisuke Nakane, Takashiro Akitsu, Takanori Nakane, Akihiro Kawamoto, Genji Kurisu

**Affiliations:** a Department of Chemistry, Faculty of Science, Tokyo University of Science, 1-3 Kagurazaka, Shinjuku-ku, Tokyo 162-8601, Japan; b Institute for Protein Research, The University of Osaka, 3-2 Yamadaoka, Suita, Osaka, 565-0871, Japan; c JEOL YOKOGUSHI Research Alliance Laboratories, The University of Osaka, 1-3 Yamadaoka, Suita, Osaka, 565-0871, Japan

**Keywords:** Fe(II) complex, azo­benzene, aldehyde, crystal structure, MicroED

## Abstract

The title compound, di­aquabis­{2-formyl-6-meth­oxy-4-[(*E*)-2-phenyl­diazen-1-yl]phenolato-κ^2^*O*^1^,*O*^2^}iron(II), [Fe(C_14_H_11_N_2_O_3_)_2_(H_2_O)_2_], comprises two bi­dentate ligands derived from 2-meth­oxy-4-(phenyl­diazen­yl)-6-formyl­phenol and two coordinated water mol­ecules. The Fe^II^ center is located at a crystallographic inversion center and adopts an octa­hedral coordination geometry. Both azo-phenolate ligands coordinate in a bidentate chelating mode (κ^2^*O*,*O*′) through the phenolate oxygen and formyl oxygen atoms in a centrosymmetric arrangement. The crystal structure was determined at 79 K using the MicroED method (λ = 0.02508 Å). The complex crystallizes in the monoclinic space group *P*2_1_/*n* with *Z* = 2. The azo groups adopt *trans* conformations. A Hirshfeld surface analysis indicates that the most important contributions to the packing are from H⋯H (36.8%) and C⋯H/H⋯C (31.9%) contacts.

## Abstract 2

The title compound, di­aquabis­{2-formyl-6-meth­oxy-4-[(*E*)-2-phenyl­diazen-1-yl]phenolato-κ^2^*O*^1^,*O*^2^}iron(II), [Fe(C_14_H_11_N_2_O_3_)_2_(H_2_O)_2_], comprises two bidentate ligands derived from 2-meth­oxy-4-(phenyl­diazen­yl)-6-formyl­phenol and two coordinated water mol­ecules. The crystal structure was determined at 79 K using the MicroED method (λ = 0.02508 Å).

## 1. Chemical context

Vanillin (4-hy­droxy-3-meth­oxy­benzaldehyde) and its derivatives are commercially available and biocompatible; because they possess both phenolic hydroxyl groups and aldehyde functional groups, they serve as versatile building blocks for the construction of multidentate ligands (Andruh, 2015 ▸). The meth­oxy substituent on the aromatic ring influences the electronic properties through electron-donating effects, thereby modulating the redox potential and coordination behaviour of the resulting metal complexes (Yamane *et al.*, 2017 ▸; Soni *et al.*, 2020 ▸; Kashiwagi *et al.*, 2019 ▸). The synthesis of azo-Schiff base hybrid ligands combining azo groups with vanillin-derived moieties represents an intriguing approach for the development of compounds that unite the coordination versatility of Schiff bases with the chromophoric properties of azo functionalities.

In our laboratory, we have been investigating metal complexes with multifunctional ligands for potential applications in dye-sensitized solar cells, flame retardants in heat-stabilized materials, and artificial metalloenzymes (Yamane *et al.*, 2017 ▸; Soni *et al.*, 2020 ▸; Kashiwagi *et al.*, 2019 ▸). Metal–salen complexes, particularly those incorporating azo-functionalized building blocks combined with chiral di­amine moieties such as (1*R*,2*R*)-(+)-1,2-di­phenyl­ethyl­enedi­amine, have attracted considerable inter­est due to their potential as asymmetric catalysts and chiral recognition materials.

Structural characterization of metal complexes bearing salicylaldehyde-based ligands remains limited in the literature (Akitsu *et al.*, 2005*a* ▸,*b* ▸; Watanabe *et al.*, 2009 ▸). The title compound, [Fe(C_14_H_11_N_2_O_3_)_2_(H_2_O)_2_], is a highly symmetric complex centred on an Fe^II^ ion, which was unexpectedly obtained as a side product of microcrystalline powder during synthesis. In this report, we describe the crystal structure of this Fe^II^ complex, whose structure was determined using microcrystal electron diffraction (MicroED).
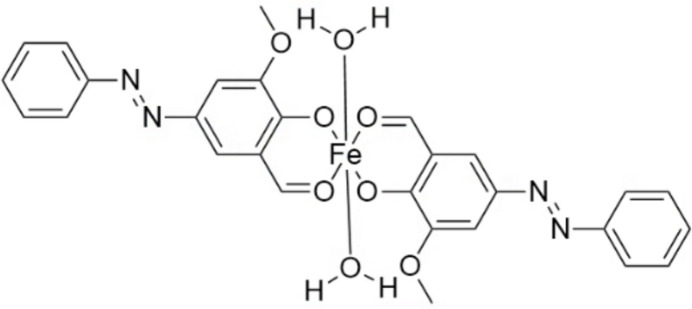


## 2. Structural commentary

**Figure 1:**
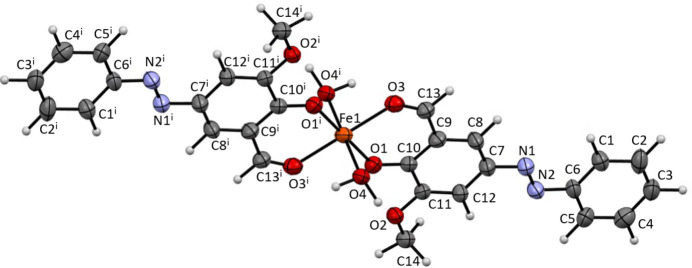
Mol­ecular structure of the title compound with the atom labelling. Displacement ellipsoids are drawn at the 50% probability level [symmetry code: (i) −*x*, 1 − *y*, −*z*].

The complex mol­ecule (Fig. 1 ▸) crystallizes in the monoclinic space group *P*2_1_/*n* with atom Fe1 positioned at a crystallographic inversion center, forming a symmetric pseudo-octa­hedral structure. The Fe1 center is six-coordinate with an O_6_ donor set, comprising oxygen atoms from two deprotonated ones (O1, O1^i^), two formyl groups (O3, O3^i^) and two coordinated water mol­ecules (O4, O4^i^). The resulting coordination geometry affords a *trans*-octa­hedral arrangement.

The principal Fe—O bond distances are: Fe1—O1 (phenolate) = 1.906 (3) Å, Fe1—O3 (form­yl) = 2.029 (3) Å, and Fe1—O4 (water) = 2.109 (4) Å. These bond lengths are consistent with those typically observed in six-coordinate Fe^II^ complexes with mixed oxygen donor atoms. Each 2-meth­oxy-4-(phenyl­diazen­yl)-6-formyl­phenolato ligand coordinates in a bidentate chelating mode (κ^2^*O*,*O*′) through the deprotonated phenolate oxygen (O1) and formyl oxygen (O3) atoms. The chelate bite angle O1—Fe1—O3 is 86.20 (14)°, indicating a small distortion from the ideal octa­hedral angle of 90° (Table 1 ▸).

The azo group (N1=N2) within the ligand exhibits a bond distance of 1.185 (6) Å, characteristic of a strong double bond, and adopts a *trans* conformation. The conjugated ligand framework contributes to the rigidity and near-planarity of the aromatic system. Due to the centrosymmetric nature of the complex, the asymmetric unit contains exactly half of the mol­ecule, with the complete structure generated by inversion symmetry.

## 3. Supra­molecular features

The crystal structure of the title compound is consolidated by dense packing and diverse non-covalent inter­actions. Hydrogen bonding plays a crucial role in the supra­molecular assembly: the coordinated water mol­ecules (O4) act as hydrogen-bond donors to adjacent formyl oxygen atoms (O3) and, to a lesser extent, to meth­oxy oxygen atoms (O2) (Table 2 ▸). The O4—H1*b*⋯O3 hydrogen bond propagates along the *a-*axis direction via crystallographic translation, linking the mol­ecules into a one-dimensional chain parallel to the *a* axis.

Hirshfeld surface analysis (Spackman & Jayatilaka, 2009 ▸; McKinnon *et al.*, 2007 ▸) was performed to qu­anti­tatively characterize the inter­molecular inter­actions. As shown in the Hirshfeld surface analysis, C—H⋯π inter­actions between aromatic C—H groups and the π-electron systems of neighbouring rings contribute significantly to the crystal packing efficiency. Additional weak C—H⋯O hydrogen bonds between aromatic C—H groups and coordinated oxygen atoms further consolidate the structure.

The relative contributions to the total Hirshfeld surface are: H⋯H contacts (36.8%), C⋯H/H⋯C contacts (31.9%), O⋯H/H⋯O contacts (18.7%), and C⋯C contacts (1.7%). The high proportion of H⋯H inter­actions reflects efficient van der Waals packing and space filling in the crystal structure. The substantial C⋯H/H⋯C contribution arises primarily from C—H⋯π inter­actions involving the extensive aromatic ring systems present in the ligand framework. The moderate O⋯H/H⋯O contribution (18.7%) corresponds to both O—H⋯O and C—H⋯O hydrogen bonds linking adjacent mol­ecules. The relatively low C⋯C contribution (1.7%) indicates minimal face-to-face π–π stacking, with the crystal packing instead dominated by edge-to-face or T-shaped aromatic inter­actions.

**Figure 2:**
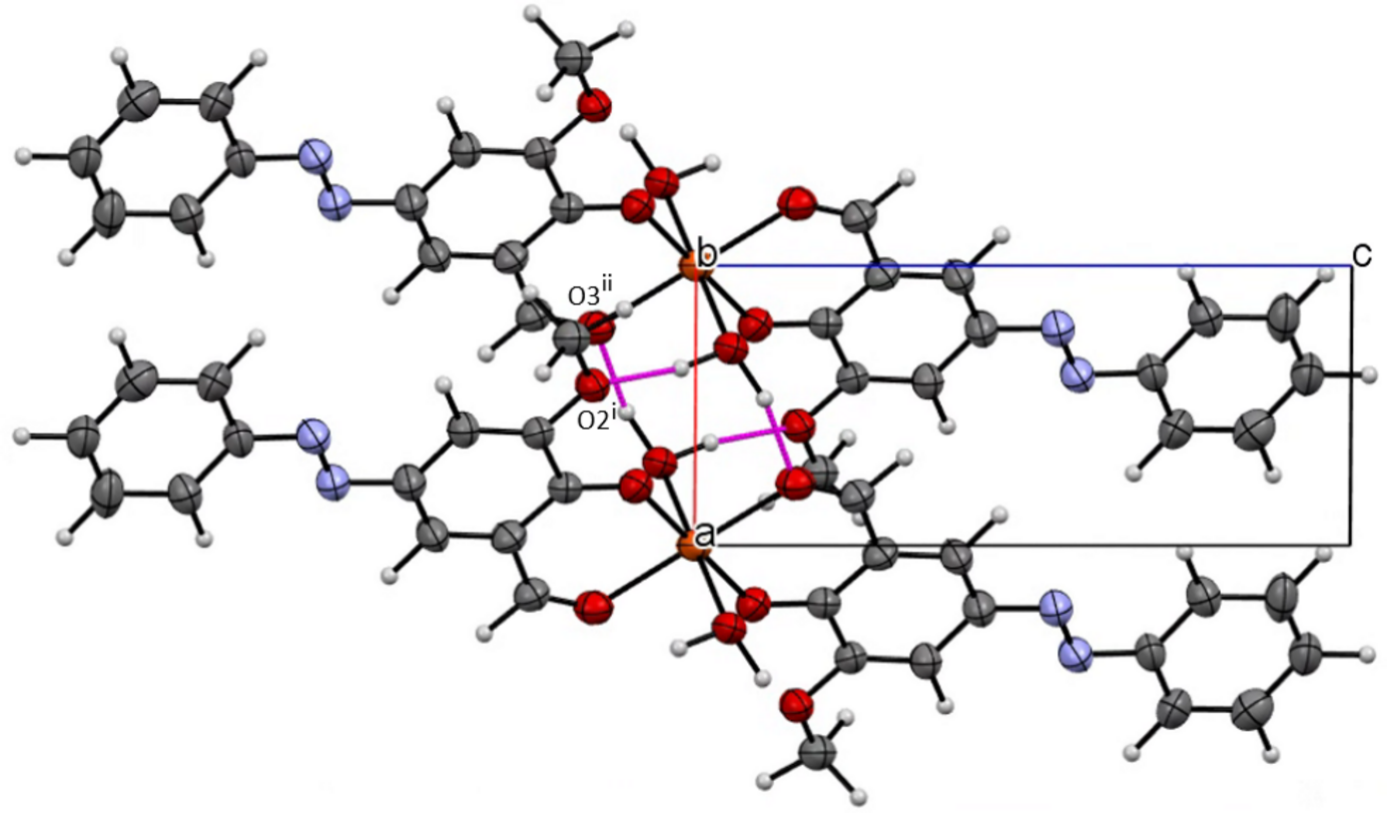
Crystal packing of the title compound viewed down from the crystallographic *b* axis. Lines indicate inter­molecular hydrogen bonds. [Symmetry codes: (i) −*x*, 1 − *y*, −*z*; (ii) −*x* + 1, −*y* + 1, −*z*].

**Figure 3:**
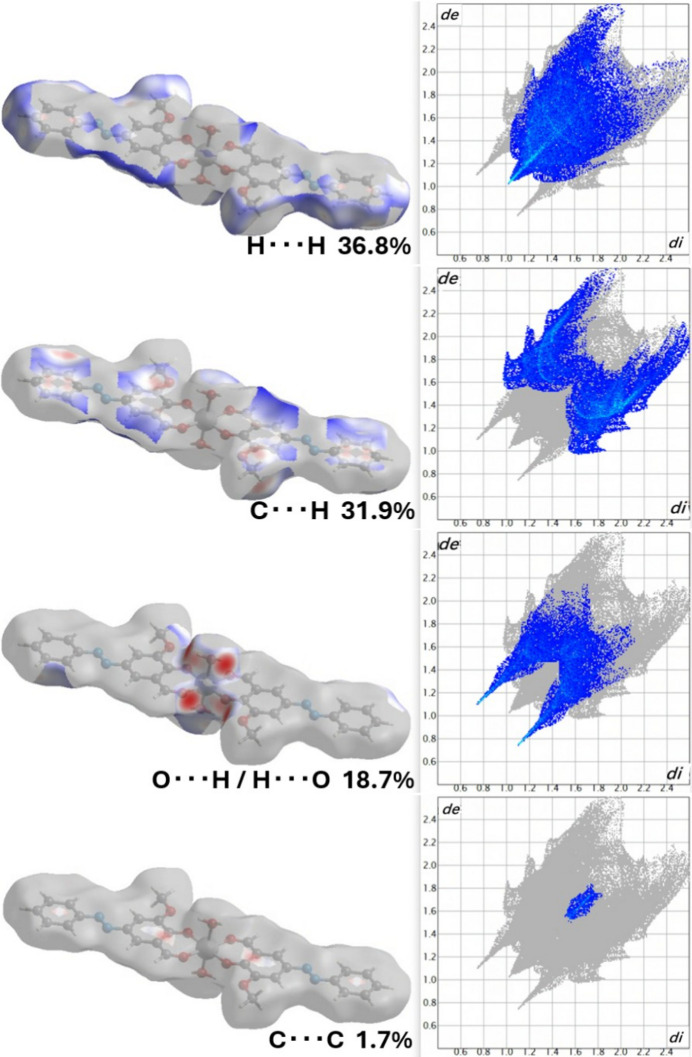
Hirshfeld surfaces mapped over *d*_norm_ and two-dimensional fingerprint plots.

The inter­molecular C—H⋯O hydrogen bonds are visualized as red spots near O3 and O1 on the Hirshfeld surfaces mapped over *d*_norm_ (Fig. 2 ▸, Fig. 3 ▸). The O—H⋯O hydrogen bonds are the dominant inter­molecular inter­actions, and other weak inter­actions such as C—H⋯π and C—H⋯O are of minor importance. The enrichment ratios are EHC = 1.32, ENH = 1.41, EHH = 0.83, ECC = 0.52 and EOH = 1.36 (Jelsch *et al.*, 2014 ▸).

## 4. Database survey

A survey of the Cambridge Structural Database (CSD, Version 5.43, update November 2024; Groom *et al.*, 2016 ▸) revealed several reported iron complexes containing azo groups with the metal ions in both +2 and +3 oxidation states. While mononuclear complexes of the general formula [Fe(*L*)_2_(H_2_O)_2_] are documented, complexes bearing azo-vanillin frameworks similar to the title compound remain relatively uncommon.

Fe—O bond distances in Fe^II^ complexes typically range from 1.95 to 2.15 Å for phenolate coordination and 2.05 to 2.25 Å for neutral oxygen donors such as water or aldehyde groups, while the corresponding distances in Fe^III^ complexes are typically shorter at 1.85-2.00 Å for phenolate and 1.95-2.10 Å for neutral donors, reflecting the larger ionic radius of Fe^II^ compared to Fe^III^. The Fe—O bond lengths observed in the title complex fall within these established ranges for Fe^II^ complexes, supporting the structural validity of the determined model despite the challenges inherent in MicroED data collection and refinement. Related structures include Fe^II^ complexes with salicylaldimine ligands and azo-containing Schiff base complexes (Keypour *et al.*, 2013 ▸), which exhibit similar octa­hedral coordination geometries with mixed O/N donor sets.

The presence of coordinated water mol­ecules in six-coordinate Fe^II^ complexes is common when the primary ligands provide fewer than six donor atoms. The *trans* arrangement of water ligands in the title compound represents a frequently observed configuration in octa­hedral metal complexes, providing charge balance and completing the coordination sphere.

## 5. Synthesis and crystallization

While the intended product was an iron–salen complex incorporating di­amine, ^1^H NMR and MicroED analysis revealed that the target complex was not formed. Instead, an unexpected bis-(azo-vanillinato)Fe^II^ complex [Fe(C_14_H_11_N_2_O_3_)_2_(H_2_O)_2_] was obtained.

The original preparation procedures are as follows. Aniline (0.311 mL, 0.3 mmol) was dissolved in 6 *M* of hydro­chloric acid (3 mL) and cooled in an ice bath. An aqueous solution of sodium nitrite (NaNO_2_, 0.023 g, 0.33 mmol in 2 mL water) was added dropwise at 273–278 K to generate the diazo­nium salt. After stirring for 30 min, *o*-vanillin (0.046 g, 0.3 mmol) in ethanol (5 mL) was added and stirred for 1 h at ice-bath temperature. The pH was adjusted to approximately 10 by addition of 10% aqueous sodium hydroxide solution, and the precipitated azo-vanillin was collected by suction filtration.

The azo-vanillin inter­mediate was dissolved in ethanol (10 mL), and (1*R*,2*R*)-(+)-1,2-di­phenyl­ethyl­enedi­amine (0.032 g, 0.15 mmol) in ethanol (5 mL) was added and stirred at 313 K for 3 h. Fe^II^ sulfate hepta­hydrate (FeSO_4_·7H_2_O, 0.021 g, 0.075 mmol) in water (2 mL) was added and stirred at 313 K for 2 h to give a dark-brown solution with precipitates, which were filtered, dried and subjected to MicroED

## 6. Refinement

**Figure 4:**
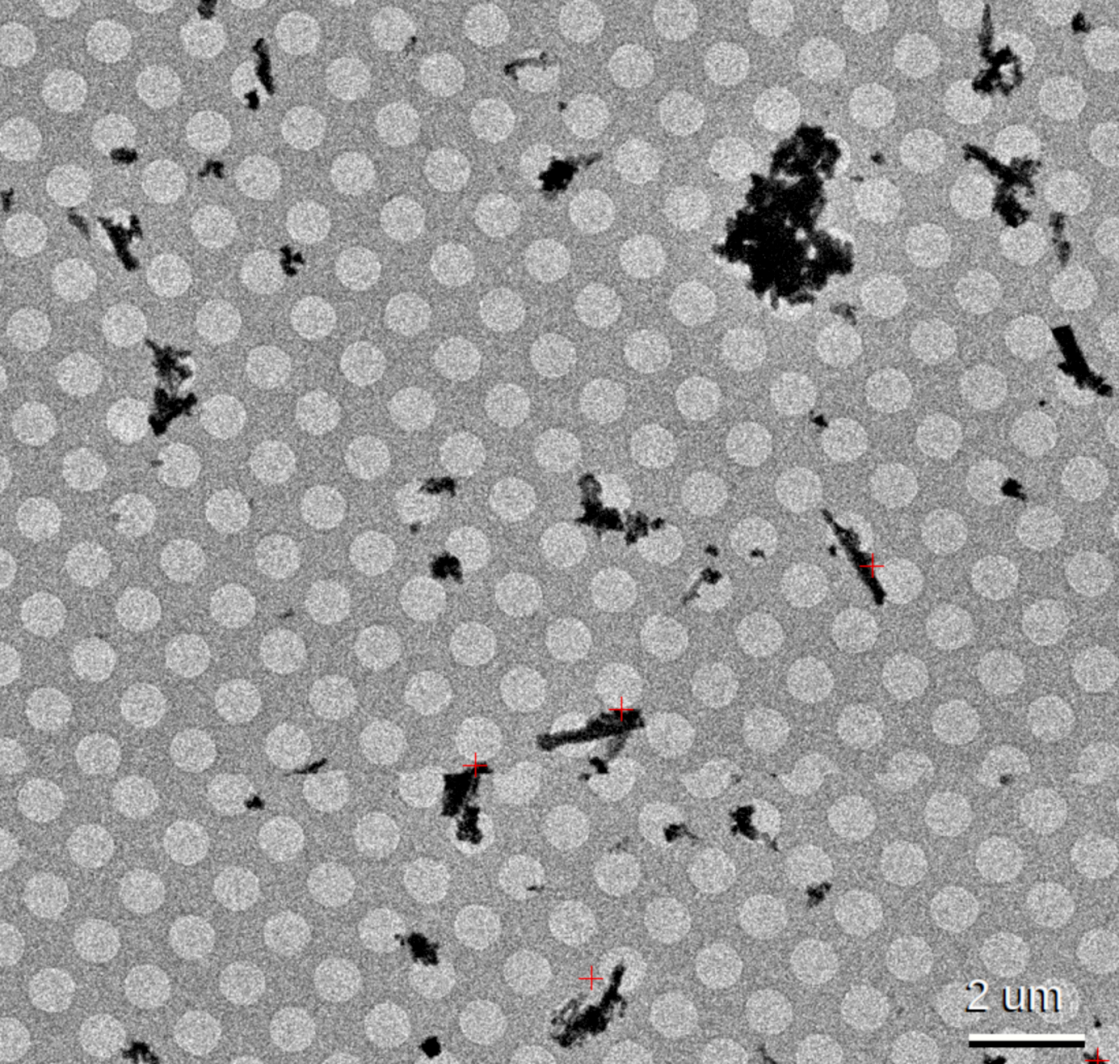
SerialEM square montage of the MicroED grid: The red crosses indicate positions screened for diffraction. Discontinuities in the image are due to alignment errors in the montaging process.

**Figure 5:**
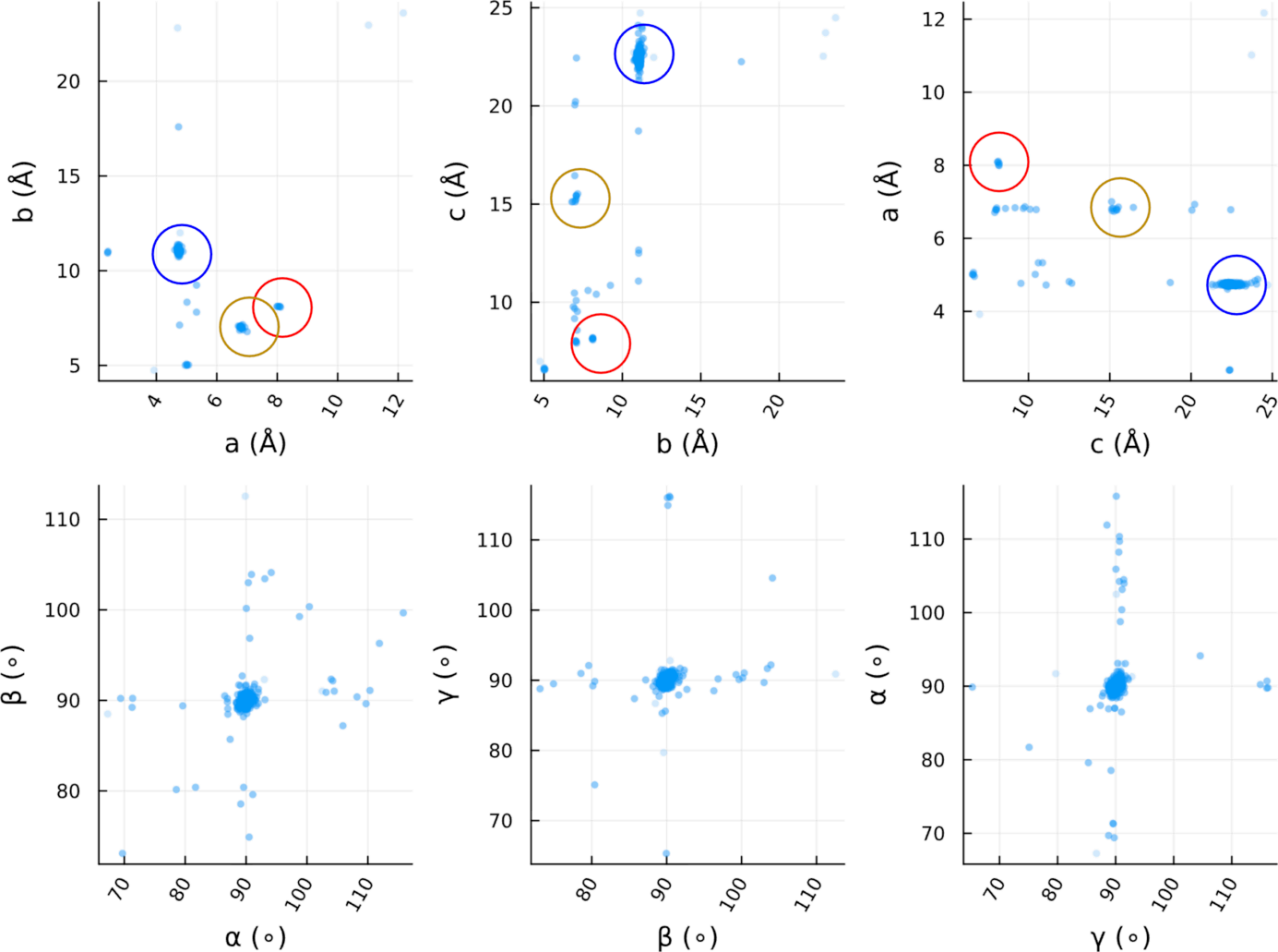
Scatter plots of unit-cell parameters: 298 crystals that diffracted to better than 1.2 Å were plotted out of 377 measured positions. This is a raw result before applying prior cell information and Bravais lattice constraints. The plot includes low-quality and/or mis-indexed crystals that were rejected in later steps of data processing. The blue cluster corresponds to the Fe complex described in this study. The red cluster was the free ligand and the orange cluster was an unknown inorganic salt.

Crystal data, data collection, and structure refinement details are summarized in Tables 3 ▸ and 4 ▸. MicroED data were collected at 79 K on a Talos Arctica electron microscope equipped with a Falcon 3 direct electron detector, controlled by *SerialEM* (Mastronarde, 2003 ▸) (Table 7 and Fig. 4 ▸). The diffraction patterns were processed with *DIALS* (Winter *et al.*, 2018 ▸; Clabbers *et al.*, 2018 ▸) and *xia2.multiplex* (Gildea *et al.*, 2022 ▸), parallelized by GNU parallel (Tange, 2011 ▸). Data scaling was performed using *dials.scale* (Beilsten-Edmands *et al.*, 2020 ▸). Crystallographic merging statistics are shown in Tables 5 ▸, 6 ▸ and 7 ▸ ▸. The structure was solved by charge flipping using *olex2. solve* (Bourhis *et al.*, 2015 ▸) and kinematically refined by full-matrix least-squares procedures on *F*^2^ using *olex2.refine* (Bourhis *et al.*, 2015 ▸). All non-hydrogen atoms were refined anisotropically. In addition to the Fe–salen complex described in this manuscript, the grid contained two more components (see scatter plots of unit-cell parameters in Fig. 5 ▸). They were separately processed and phased as in Gogoi *et al.* (2023 ▸). The two components turned out to be a free ligand (CCDC-2524690; COD-3000633) and an inorganic salt (supplementary Fig. S1). Because of the very low occurrences of their crystals, their completeness was limited. Moreover, the inorganic salt could not be identified due to the difficulty in element identification by MicroED. Therefore, we do not describe their structures further in this report.

The benzene ring (C1–C6) collapsed (long C—C) during the calculation, so we used the AFIX66 constraint. Hydrogen atoms bound to carbon and oxygen were placed at peak positions and refined using a riding model.

The final reliability indices are *R*_1_ = 0.2176 [for 3421 reflections with *I* ≥ 2σ(*I*)] and *wR*_2_ = 0.5343 (all 5188 data), with a goodness-of-fit of 2.0002. The relatively high *R*-factors are typical for MicroED data and are attributed to the neglect of dynamical diffraction, partial charges and bond polarization, Additionally, the equivalent reflections showed a relatively high *R*_int_ value of 0.2335. This is also common in high-multiplicity MicroED datasets. First, intensities of equivalent reflections vary due to multiple scattering. Next, *R*_int_ increases with the multiplicity of the dataset, as pointed out in Diederichs & Karplus (1997 ▸) for the case of a related metric *R*_merge_. Despite these refinement challenges, the structural model is chemically reasonable, with the connectivity and overall mol­ecular geometry unambiguously determined. The coordination environment of the iron center and the arrangement of the azo-vanillin ligands are clearly resolved, providing valuable insight into the coordination chemistry of this unexpected product.

Readers should be aware that the estimated standard deviations (ESDs) of refined parameters are severely underestimated in MicroED. They are calculated from the covariance matrix via error-propagation by the least square refinement engine. However, necessary assumptions (independent, zero mean, random errors with known sigmas) do not hold in MicroED. For example, we estimate up to 0.3% of errors are possible in the virtual camera distance of our scope. Neglect of dynamical scattering and partial charges introduces systematic errors. Refined parameters and ESDs in the accompanying tables were automatically extracted from the refined CIF file as is but we consider numbers beyond the second decimal place as qu­anti­tatively dubious. Indeed, recent analysis by Gemmi *et al.* (2026 ▸) suggested that an average accuracy of atomic positions achieved though kinematic refinement is about 0.03–0.05 Å depending on the beam sensitivity of the sample.

## 7. Data Availability

The refined coordinates of the Fe complex (CCDC-2552645; COD-3000632) and the free ligand (CCDC-2524690; COD-3000633) have been deposited at the Cambridge Crystallographic Data Centre and the Crystallography Open Database. The raw diffraction images have been deposited to XRDa-469(https://doi.org/10.51093/xrd-00469). Scripts and manuals for MicroED data collection and processing are available at our GitHub repository https://github.com/GKLabIPR/MicroED.

## Supplementary Material

Crystal structure: contains datablock(s) I. DOI: 10.1107/S2056989026004755/meu2001sup1.cif

Structure factors: contains datablock(s) I. DOI: 10.1107/S2056989026004755/meu2001Isup2.hkl

Structure of the free ligand contained in the MicroED grid. DOI: 10.1107/S2056989026004755/meu2001sup3.tif

Structure of an unknown salt contained in the MicroED grid. Because of limited data completeness and the difficulty of element identification in MicroED, we could not confidently identify the salt and finalize the refinement. DOI: 10.1107/S2056989026004755/meu2001sup4.tif

Intermolecular C---H...O hydrogen bonds as red spots. DOI: 10.1107/S2056989026004755/meu2001sup5.tif

CCDC reference: 2552645

Additional supporting information:  crystallographic information; 3D view; checkCIF report

## Figures and Tables

**Table 1:** Selected geometric parameters (Å, °)

Fe1—O1	1.906 (3)	O1—C10	1.222 (4)
Fe1—O3	2.029 (3)	O3—C13	1.147 (5)
Fe1—O4	2.109 (4)	N2—N1	1.185 (6)
			
O3—Fe1—O1	86.20 (14)	C14—O2—C11	116.7 (3)
O4—Fe1—O1	88.32 (15)	H1*b*—O4—H1*a*	104.5
O4—Fe1—O3	90.41 (13)	C7—N1—N2	116.5 (4)
C10—O1—Fe1	130.0 (3)	C6—N2—N1	115.2 (3)

**Table 2:** Hydrogen-bond geometry (Å, °)

*D*—H⋯*A*	*D*—H	H⋯*A*	*D*⋯*A*	*D*—H⋯*A*
O4—H1*a*⋯O2^i^	1.04	1.82	2.845	169
O4—H1*b*⋯O3^ii^	1.04	1.82	2.788	153

Hydrogen-atom positions cannot be accurately determined in kinematical refinement of MicroED datasets. Therefore, distances involving hydrogen atoms are not quantitatively reliable and s.u.’s are not estimated. Symmetry codes: (i) 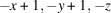; (ii) 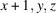.

**Table 3:** Experimental details

Crystal data	
Chemical formula	[Fe(C_14_H_11_N_2_O_3_)_2_(H_2_O)_2_]
*M*_r_	602.38
Crystal system, space group	Monoclinic, *P*2_1_/*n*
Temperature (K)	79
*a*, *b*, *c* (Å)	4.7419 (4), 22.447 (6), 11.0960 (11)
β (°)	90.396 (8)
*V* (Å^3^)	1181.0 (3)
*Z*	2
Radiation type	Electron, λ = 0.02508 Å
μ (mm^−1^)	0.00
Crystal size (mm)	0.02 × 0.0002 × 0.0002

Data collection	
Diffractometer	Talos Arctica electron microscope
No. of measured, independent and observed [*I* ≥ 2u(*I*)] reflections	277706, 5188, 3421
*R*_int_	0.234
(sin θ/λ)_max_ (Å^−1^)	0.807

Refinement	
*R*[*F*^2^ > 2σ(*F*^2^)], *wR*(*F*^2^), *S*	0.218, 0.534, 2.00
No. of reflections	5188
No. of parameters	178
H-atom treatment	H-atom parameters constrained
Δρ_max_, Δρ_min_	1.68, −0.62

Density values in MicroED maps are proportional to Coulomb potential but the scale is not necessarily absolute. Computer programs: *OLEX2.solve* (Bourhis *et al.*, 2015 ▸), *OLEX2.refine* (Bourhis *et al.*, 2015 ▸), *OLEX2* (Dolomanov *et al.*, 2009 ▸) and *publCIF* (Westrip, 2010 ▸).

**Table 4:** Summary of MicroED data collection and processing

Microscope parameters	
Scope	Talos Arctica
Acceleration voltage	200 kV (∼0.025079 Å)
Probe	Nanoprobe mode
Gun lens	8
Beam convergence	Parallel
Beam diameter	∼1.6 um
Fluence	∼0.055 e Å^−1^ sec^−1^
Rotation	∼0.4354°/frame
Energy filtering	Not equipped
Detector	Falcon 3 (integrating mode)
Pixel size	28 µm (after binning by 2)
Camera length	615.5 mm
Frame rate	∼0.35 sec / fraction
Microscope control	SerialEM
Stage temperature	∼79 K
	
Data collection and processing	
Sample grid	Qu­antiFoil Mo R0.6/1.0
Snapshot screening	> 750 positions
Data collection	377 positions
Initial indexing success	312 crystals
Processing	*DIALS* with GNU parallel
	
Cluster 1	(blue cluster; the Fe complex)
Indexed	241 crystals
Selected for merging	
Selection criteria	CC1/2@0.72Å > 1/2, *xia.multiplex*, *dials.scale* with deltacchalf filtering
	
Cluster 2	(red cluster; the free ligand)
Indexed	5 crystals
Selected for merging	5 crystals
Selection criteria	none (used all)
	
Cluster 3	(orange cluster; unknown inorganic salt)
Indexed	7 crystals
Selected for merging	7 crystals
Selection criteria	none (used all)

**Table 5:** Merging statistics for the Fe complex

*d*_max_	*d*_min_	obs	uniq	mult.	%comp	<*I*/σ(*I*)>	*r*_pim_	*cc*1/2
6.20	1.67	11334	275	41.21	99.28	49.7	0.020	0.984*
1.67	1.33	13981	270	51.78	100.00	38.0	0.021	0.992*
1.33	1.16	13115	268	48.94	100.00	30.4	0.025	0.995*
1.16	1.06	15612	277	56.36	100.00	25.2	0.028	0.997*
1.06	0.98	12292	252	48.78	100.00	20.1	0.034	0.995*
0.98	0.93	13964	260	53.71	100.00	17.9	0.034	0.991*
0.93	0.88	16465	284	57.98	100.00	13.3	0.048	0.958*
0.88	0.84	13495	260	51.90	100.00	9.9	0.059	0.964*
0.84	0.81	11891	253	47.00	100.00	6.9	0.080	0.932*
0.81	0.78	14647	266	55.06	100.00	6.5	0.076	0.923*
0.78	0.76	16342	269	60.75	100.00	5.3	0.090	0.914*
0.76	0.73	15344	273	56.21	100.00	4.5	0.109	0.903*
0.73	0.72	13776	262	52.58	100.00	3.5	0.133	0.882*
0.72	0.70	12463	252	49.46	100.00	2.4	0.192	0.633*
0.70	0.68	12810	257	49.84	100.00	2.5	0.191	0.840*
0.68	0.67	14628	255	57.36	100.00	2.4	0.184	0.693*
0.67	0.65	17458	278	62.80	100.00	2.9	0.154	0.797*
0.65	0.64	15599	279	55.91	100.00	1.7	0.273	0.464*
0.64	0.63	14029	269	52.15	100.00	1.5	0.284	0.552*
0.63	0.62	14499	263	55.13	100.00	1.6	0.278	0.618*
6.20	0.62	283744	5322	53.32	99.96	12.4	0.033	0.995*

**Table 6:** Merging statistics for the free ligand

*d*_max_	*d*_min_	obs	uniq	mult.	%comp	<*I*/σ(*I*)>	*r*_pim_	*cc*1/2
5.55	1.95	181	62	2.92	80.52	27.5	0.049	0.995*
1.95	1.56	247	74	3.34	89.16	20.2	0.060	0.979*
1.56	1.36	203	59	3.44	89.39	11.2	0.105	0.920*
1.36	1.24	268	70	3.83	89.74	9.3	0.110	0.964*
1.24	1.15	204	60	3.40	90.91	9.4	0.107	0.956*
1.15	1.08	295	77	3.83	92.77	9.6	0.116	0.932*
1.08	1.03	201	58	3.47	87.88	9.5	0.112	0.956*
1.03	0.99	277	71	3.90	93.42	7.3	0.135	0.965*
0.99	0.95	254	68	3.74	86.08	4.9	0.153	0.892*
0.95	0.92	221	63	3.51	92.65	4.4	0.201	0.815*
0.92	0.89	257	64	4.02	88.89	3.5	0.215	0.693*
0.89	0.86	283	73	3.88	90.12	2.9	0.218	0.700*
0.86	0.84	242	63	3.84	92.65	2.1	0.288	0.517*
0.84	0.82	205	59	3.47	86.76	2.3	0.275	0.706*
0.82	0.80	269	70	3.84	88.61	1.8	0.388	0.665*
5.55	0.80	3607	991	3.64	89.20	8.4	0.113	0.973*

**Table 7:** Merging statistics for the unknown inorganic salt

*d*_max_	*d*_min_	obs	uniq	mult.	%comp	<*I*/σ(*I*)>	*r*_pim_	*cc*1/2
4.89	1.59	691	110	6.28	82.09	17.4	0.074	0.983*
1.59	1.27	719	103	6.98	89.57	12.5	0.088	0.971*
1.27	1.11	739	102	7.25	89.47	8.4	0.114	0.944*
1.11	1.01	765	96	7.97	90.57	8.2	0.120	0.973*
1.01	0.94	824	105	7.85	90.52	5.6	0.117	0.885*
0.94	0.88	662	88	7.52	88.00	3.9	0.181	0.833*
0.88	0.84	841	98	8.58	91.59	4.0	0.151	0.864*
0.84	0.80	759	99	7.67	92.52	3.5	0.167	0.684*
0.80	0.77	731	94	7.78	91.26	2.5	0.186	0.859*
0.77	0.74	815	99	8.23	91.67	2.3	0.231	0.723*
0.74	0.72	819	101	8.11	90.99	2.2	0.214	0.482*
0.72	0.70	610	80	7.62	89.89	1.9	0.340	0.763*
0.70	0.68	889	100	8.89	90.91	1.5	0.266	0.641*
0.68	0.67	777	99	7.85	92.52	1.4	0.279	0.542*
0.67	0.65	724	93	7.78	89.42	1.2	0.403	0.514*
4.89	0.65	11365	1467	7.75	90.00	5.3	0.112	0.981*

## supplementary crystallographic information

### Diaquabis{2-formyl-6-methoxy-4-[(E)-2-phenyldiazen-1-yl]phenolato-κ2O1,O2}iron(II) Crystal data

**Table:**

[Fe(C_14_H_11_N_2_O_3_)_2_(H_2_O)_2_]	*F*(000) = 231.684
*M**_r_* = 602.38	*D*_x_ = 1.694 Mg m^−^^3^
Monoclinic, *P*2_1_/*n*	Electron radiation, λ = 0.02508 Å
*a* = 4.7419 (4) Å	Cell parameters from 16876 reflections
*b* = 22.447 (6) Å	θ = 6.6–56.6°
*c* = 11.0960 (11) Å	µ = 0.000 mm^−^^1^
β = 90.396 (8)°	*T* = 79 K
*V* = 1181.0 (3) Å^3^	Powder, orange
*Z* = 2	0.01 × 0.001 × 0.001 mm

### Diaquabis{2-formyl-6-methoxy-4-[(E)-2-phenyldiazen-1-yl]phenolato-κ2O1,O2}iron(II) Data collection

**Table:**

Talos Arctica electron microscope diffractometer	*R*_int_ = 0.234
Radiation source: Talos Arctica Field Emission Gun	θ_max_ = 1.2°, θ_min_ = 0.1°
continuous rotation electron diffraction (MicroED) scans	*h* = −7→7
277706 measured reflections	*k* = −36→36
5188 independent reflections	*l* = −17→17
3421 reflections with *I*≥ 2u(*I*)	

### Diaquabis{2-formyl-6-methoxy-4-[(E)-2-phenyldiazen-1-yl]phenolato-κ2O1,O2}iron(II) Refinement

**Table:**

Refinement on *F*^2^	24 constraints
Least-squares matrix: full	Primary atom site location: iterative
*R*[*F*^2^ > 2σ(*F*^2^)] = 0.218	H-atom parameters constrained
*wR*(*F*^2^) = 0.534	*w* = 1/[σ^2^(*F*_o_^2^) + (0.2*P*)^2^] where *P* = (*F*_o_^2^ + 2*F*_c_^2^)/3
*S* = 2.00	(Δ/σ)_max_ = 0.001
5188 reflections	Δρ_max_ = 1.68 e Å^−^^3^
178 parameters	Δρ_min_ = −0.62 e Å^−^^3^
0 restraints	

### Diaquabis{2-formyl-6-methoxy-4-[(E)-2-phenyldiazen-1-yl]phenolato-κ2O1,O2}iron(II) Fractional atomic coordinates and isotropic or equivalent isotropic displacement parameters (Å^2^)

**Table:**

	*x*	*y*	*z*	*U*_iso_*/*U*_eq_	
Fe1	0.0	0.5	0.0	0.0396 (7)	
O1	0.2119 (6)	0.55697 (19)	0.0903 (3)	0.0427 (10)	
O2	0.5723 (6)	0.63117 (19)	0.1573 (3)	0.0409 (9)	
O3	−0.2221 (6)	0.48661 (18)	0.1532 (3)	0.0423 (10)	
O4	0.2951 (6)	0.4348 (2)	0.0538 (3)	0.0448 (10)	
H1a	0.367 (3)	0.4130 (6)	−0.0228 (3)	0.0673 (15)*	
H1b	0.476 (2)	0.4423 (2)	0.1053 (13)	0.0673 (15)*	
N1	0.2246 (7)	0.6181 (2)	0.5519 (3)	0.0462 (11)	
N2	0.3901 (7)	0.6548 (2)	0.5823 (3)	0.0464 (11)	
C1	0.1835 (5)	0.64836 (14)	0.7774 (2)	0.0469 (12)	
H1	0.0233 (5)	0.61580 (14)	0.7478 (2)	0.0562 (14)*	
C2	0.1866 (6)	0.66845 (16)	0.8959 (2)	0.0587 (14)	
H2	0.0288 (6)	0.65182 (16)	0.9603 (2)	0.0704 (17)*	
C3	0.3885 (6)	0.70949 (16)	0.93334 (18)	0.0563 (14)	
H3	0.3909 (6)	0.72543 (16)	1.02738 (18)	0.0675 (17)*	
C4	0.5874 (6)	0.73045 (15)	0.8522 (2)	0.0539 (13)	
H4	0.7476 (6)	0.76301 (15)	0.8819 (2)	0.0647 (16)*	
C5	0.5843 (5)	0.71036 (16)	0.7337 (2)	0.0518 (13)	
H5	0.7421 (5)	0.72699 (16)	0.6694 (2)	0.0621 (16)*	
C6	0.3824 (5)	0.66932 (15)	0.69635 (17)	0.0433 (12)	
C7	0.2252 (8)	0.6029 (2)	0.4355 (3)	0.0461 (12)	
C8	0.0401 (8)	0.5627 (2)	0.3995 (3)	0.0461 (12)	
H6	−0.1088 (8)	0.5436 (2)	0.4650 (3)	0.0553 (14)*	
C9	0.0305 (8)	0.5446 (2)	0.2835 (3)	0.0422 (11)	
C10	0.2059 (7)	0.5688 (2)	0.1977 (3)	0.0366 (10)	
C11	0.4016 (7)	0.6108 (2)	0.2396 (3)	0.0392 (11)	
C12	0.4135 (8)	0.6268 (2)	0.3536 (3)	0.0396 (10)	
H7	0.5731 (8)	0.6594 (2)	0.3841 (3)	0.0475 (12)*	
C13	−0.1757 (8)	0.5049 (2)	0.2477 (3)	0.0414 (11)	
H8	−0.3149 (8)	0.4887 (2)	0.3198 (3)	0.0497 (13)*	
C14	0.7404 (8)	0.6756 (2)	0.1895 (4)	0.0474 (12)	
H1c	0.844 (3)	0.6937 (5)	0.1094 (4)	0.0711 (18)*	
H1e	0.6157 (9)	0.7107 (4)	0.2326 (14)	0.0711 (18)*	
H1d	0.901 (2)	0.6593 (3)	0.2531 (12)	0.0711 (18)*	

### Diaquabis{2-formyl-6-methoxy-4-[(E)-2-phenyldiazen-1-yl]phenolato-κ2O1,O2}iron(II) Atomic displacement parameters (Å^2^)

**Table:**

	*U*^11^	*U*^22^	*U*^33^	*U*^12^	*U*^13^	*U*^23^
Fe1	0.0288 (7)	0.0499 (15)	0.0402 (9)	0.0002 (7)	0.0003 (5)	−0.0078 (7)
O1	0.0359 (13)	0.054 (3)	0.0379 (14)	0.0004 (15)	−0.0024 (10)	−0.0081 (14)
O2	0.0352 (13)	0.051 (3)	0.0367 (14)	−0.0033 (14)	0.0038 (10)	−0.0097 (13)
O3	0.0350 (13)	0.044 (3)	0.0479 (17)	−0.0031 (14)	0.0005 (11)	−0.0039 (14)
O4	0.0304 (12)	0.066 (3)	0.0379 (14)	0.0002 (14)	0.0021 (9)	−0.0075 (14)
N1	0.0417 (16)	0.064 (3)	0.0330 (15)	−0.0064 (18)	0.0028 (11)	−0.0024 (16)
N2	0.0407 (16)	0.062 (3)	0.0369 (16)	−0.0085 (18)	0.0059 (12)	−0.0076 (17)
C1	0.049 (2)	0.055 (3)	0.0367 (19)	0.001 (2)	0.0072 (15)	−0.0046 (18)
C2	0.075 (3)	0.069 (4)	0.0314 (18)	0.017 (3)	−0.0039 (18)	−0.006 (2)
C3	0.056 (2)	0.080 (4)	0.0328 (19)	0.009 (3)	−0.0036 (16)	−0.007 (2)
C4	0.051 (2)	0.054 (4)	0.057 (3)	0.003 (2)	−0.0093 (19)	−0.001 (2)
C5	0.047 (2)	0.073 (4)	0.0352 (19)	0.003 (2)	−0.0075 (15)	0.002 (2)
C6	0.0446 (18)	0.058 (3)	0.0271 (15)	0.005 (2)	0.0047 (13)	−0.0007 (16)
C7	0.0436 (19)	0.062 (4)	0.0326 (17)	−0.009 (2)	0.0032 (14)	−0.0023 (18)
C8	0.0409 (18)	0.064 (4)	0.0333 (17)	−0.011 (2)	0.0077 (13)	−0.0089 (18)
C9	0.0348 (16)	0.051 (3)	0.0405 (19)	−0.0079 (18)	−0.0019 (13)	−0.0087 (18)
C10	0.0293 (14)	0.046 (3)	0.0341 (16)	−0.0013 (16)	0.0005 (11)	−0.0014 (16)
C11	0.0302 (14)	0.055 (3)	0.0320 (16)	0.0014 (17)	−0.0003 (11)	−0.0054 (16)
C12	0.0387 (16)	0.048 (3)	0.0323 (16)	−0.0010 (18)	0.0021 (12)	−0.0084 (16)
C13	0.0346 (16)	0.049 (3)	0.0403 (19)	−0.0044 (18)	−0.0014 (13)	−0.0049 (17)
C14	0.0360 (17)	0.062 (4)	0.044 (2)	0.000 (2)	0.0002 (14)	−0.008 (2)

### Diaquabis{2-formyl-6-methoxy-4-[(E)-2-phenyldiazen-1-yl]phenolato-κ2O1,O2}iron(II) Geometric parameters (Å, º)

**Table:**

Fe1—O1	1.906 (3)	C3—C4	1.3900
Fe1—O1^i^	1.906 (3)	C4—H4	1.1030
Fe1—O3	2.029 (3)	C5—C4	1.3900
Fe1—O3^i^	2.029 (3)	C5—H5	1.1030
Fe1—O4	2.109 (4)	C6—C1	1.3900
Fe1—O4^i^	2.109 (4)	C6—C5	1.3900
O1—C10	1.222 (4)	C7—C8	1.319 (6)
O2—C11	1.307 (5)	C7—C12	1.387 (5)
O2—C14	1.325 (6)	C8—H6	1.1030
O3—C13	1.147 (5)	C9—C8	1.350 (5)
O4—H1a	1.0400	C9—C13	1.378 (6)
O4—H1b	1.0400	C10—C9	1.380 (5)
N1—C7	1.336 (5)	C10—C11	1.399 (6)
N2—N1	1.185 (6)	C11—C12	1.316 (5)
N2—C6	1.308 (4)	C12—H7	1.1030
C1—H1	1.1030	C13—H8	1.1030
C1—C2	1.3900	C14—H1c	1.0970
C2—H2	1.1030	C14—H1e	1.0970
C3—C2	1.3900	C14—H1d	1.0970
C3—H3	1.1030		
			
O1^i^—Fe1—O1	180.0	H4—C4—C5	120.00 (8)
O3^i^—Fe1—O1	93.80 (14)	C4—C5—H5	120.00 (8)
O3—Fe1—O1	86.20 (14)	C4—C5—C6	120.0
O3—Fe1—O1^i^	93.80 (14)	H5—C5—C6	120.00 (8)
O3^i^—Fe1—O1^i^	86.20 (14)	C1—C6—N2	124.4
O3^i^—Fe1—O3	180.0	C5—C6—N2	115.5
O4—Fe1—O1	88.32 (15)	C5—C6—C1	120.0
O4—Fe1—O1^i^	91.68 (15)	C8—C7—N1	117.5 (4)
O4^i^—Fe1—O1	91.68 (15)	C8—C7—C12	119.8 (4)
O4^i^—Fe1—O1^i^	88.32 (15)	C12—C7—N1	122.7 (4)
O4^i^—Fe1—O3	89.59 (13)	H6—C8—C7	119.6 (2)
O4—Fe1—O3	90.41 (13)	H6—C8—C9	119.6 (2)
O4—Fe1—O3^i^	89.59 (13)	C9—C8—C7	120.8 (4)
O4^i^—Fe1—O3^i^	90.41 (13)	C8—C9—C10	121.5 (4)
O4—Fe1—O4^i^	180.0	C8—C9—C13	119.2 (4)
C10—O1—Fe1	130.0 (3)	C13—C9—C10	119.0 (4)
C14—O2—C11	116.7 (3)	C9—C10—O1	127.2 (4)
C13—O3—Fe1^i^	128.0 (3)	C9—C10—C11	116.0 (3)
H1a—O4—Fe1^i^	108.4	C11—C10—O1	116.8 (3)
H1b—O4—Fe1^i^	125.9	C10—C11—O2	114.6 (3)
H1b—O4—H1a	104.5	C12—C11—O2	123.6 (4)
C7—N1—N2	116.5 (4)	C12—C11—C10	121.8 (4)
C6—N2—N1	115.2 (3)	C11—C12—C7	120.0 (4)
H1—C1—C6	120.00 (8)	H7—C12—C7	120.0 (2)
C2—C1—H1	120.00 (8)	H7—C12—C11	120.0 (2)
C2—C1—C6	120.0	C9—C13—O3	128.8 (4)
H2—C2—C1	120.00 (8)	H8—C13—O3	115.6 (3)
H2—C2—C3	120.00 (8)	H8—C13—C9	115.6 (2)
C3—C2—C1	120.0	H1c—C14—O2	109.5
C2—C3—H3	120.00 (8)	H1e—C14—O2	109.5
C2—C3—C4	120.0	H1e—C14—H1c	109.5
C4—C3—H3	120.00 (8)	H1d—C14—O2	109.5
C3—C4—C5	120.0	H1d—C14—H1c	109.5
H4—C4—C3	120.00 (8)	H1d—C14—H1e	109.5
			
Fe1—O1—C10—C9	−2.6 (5)	C1—C2—C3—C4	0.0 (3)
Fe1—O1—C10—C11	176.1 (4)	C1—C6—C5—C4	0.0 (3)
Fe1—O3—C13—C9	5.2 (5)	C5—C4—C3—C2	0.0 (3)
O1—C10—C9—C8	−178.3 (5)	C5—C6—C1—C2	0.0 (3)
O1—C10—C9—C13	−4.2 (6)	C6—C1—C2—C3	0.0 (3)
O1—C10—C11—C12	180.0 (4)	C6—C5—C4—C3	0.0 (3)
O2—C11—C10—O1	−1.5 (5)	C7—N1—N2—C6	179.2 (4)
O2—C11—C10—C9	177.3 (4)	C7—C8—C9—C13	−176.5 (5)
O2—C11—C12—C7	−179.7 (5)	C9—C8—C7—C12	−0.2 (6)
O3—C13—C9—C8	176.8 (6)	C9—C10—C11—C12	−1.2 (5)
N1—N2—C6—C1	−5.3 (6)	C10—C9—C8—C7	−2.4 (5)
N1—N2—C6—C5	177.2 (4)	C10—C9—C13—O3	2.6 (6)
N1—C7—C8—C9	−179.2 (5)	C10—C11—O2—C14	172.8 (4)
N1—C7—C12—C11	−179.0 (5)	C10—C11—C12—C7	−1.2 (5)
N2—N1—C7—C8	−179.1 (5)	C11—C10—C9—C8	3.0 (5)
N2—N1—C7—C12	1.9 (6)	C11—C10—C9—C13	177.1 (4)
N2—C6—C1—C2	−177.4 (3)	C11—C12—C7—C8	2.0 (5)
N2—C6—C5—C4	177.6 (2)	C12—C11—O2—C14	−8.7 (5)

Symmetry code: (i) −*x*, −*y*+1, −*z*.

### Diaquabis{2-formyl-6-methoxy-4-[(E)-2-phenyldiazen-1-yl]phenolato-κ2O1,O2}iron(II) Hydrogen-bond geometry (Å, º)

**Table:**

*D*—H···*A*	*D*—H	H···*A*	*D*···*A*	*D*—H···*A*
O4—H1*a*···O2^ii^	1.04	1.82	2.845	169
O4—H1*b*···O3^iii^	1.04	1.82	2.788	153

Symmetry codes: (ii) −*x*+1, −*y*+1, −*z*; (iii) *x*+1, *y*, *z*.

## References

[bb1] Akitsu, T., Takeuchi, Y. & Einaga, Y. (2005*a*). *Acta Cryst.* C**61**, m324–m328. 10.1107/S0108270105013545 15997054

[bb2] Akitsu, T., Takeuchi, Y. & Einaga, Y. (2005*b*). *Acta Cryst.* E**61**, m772–m774. 10.1107/S0108270105013545 15997054

[bb3] Andruh, M. (2015). *Dalton Trans.* **44**, 16633–16653. 10.1039/c5dt02661j 26282536

[bb4] Beilsten-Edmands, J., Winter, G., Gildea, R., Parkhurst, J., Waterman, D. & Evans, G. (2020). *Acta Cryst.* D**76**, 385–399. 10.1107/S2059798320003198 PMC7137103 32254063

[bb5] Bourhis, L. J., Dolomanov, O. V., Gildea, R. J., Howard, J. A. K. & Puschmann, H. (2015). *Acta Cryst.* A**71**, 59–75. 10.1107/S2053273314022207 PMC4283469 25537389

[bb6] Clabbers, M. T. B., Gruene, T., Parkhurst, J. M., Abrahams, J. P. & Waterman, D. G. (2018). *Acta Cryst.* D**74**, 506–518. 10.1107/S2059798318007726 PMC6096487 29872002

[bb24] Diederichs & Karplus (1997). *Nature Struct. Biol.* **4**, 269–275. 10.1038/nsb0497-269 9095194

[bb7] Dolomanov, O. V., Bourhis, L. J., Gildea, R. J., Howard, J. A. K. & Puschmann, H. (2009). *J. Appl. Cryst.* **42**, 339–341.

[bb8] Gemmi, M., Palatinus, L., Boullay, P., Abrahams, J. P., Ben Meriem, A., Cordero-Oyonarte, E., Emerson Agbemeh, V., Chintakindi, H., Faye Diouf, M. D., Filipcik, P., Gemmrich Hernandéz, L., van Genderen, E., Hadermann, J., Hajizadeh, A., Jeriga, B., Kolb, U., Matinyan, S., Passuti, S., Santucci, M., Suresh, A., Pérez, O., Tai, C.-W., Vypritskaia, A., Wang, L., Xu, H. & Zou, X. (2026). *IUCrJ* **13**, 198–210. 10.1107/S205225252600045X PMC12951830 41685623

[bb9] Gildea, R. J., Beilsten-Edmands, J., Axford, D., Horrell, S., Aller, P., Sandy, J., Sanchez-Weatherby, J., Owen, C. D., Lukacik, P., Strain-Damerell, C., Owen, R. L., Walsh, M. A. & Winter, G. (2022). *Acta Cryst.* D**78**, 752–769. 10.1107/S2059798322004399 PMC9159281 35647922

[bb10] Gogoi, D., Sasaki, T., Nakane, T., Kawamoto, A., Hojo, H., Kurisu, G. & Thakuria, R. (2023). *Cryst. Growth Des.* **23**, 5821–5826.

[bb11] Groom, C. R., Bruno, I. J., Lightfoot, M. P. & Ward, S. C. (2016). *Acta Cryst.* B**72**, 171–179. 10.1107/S2052520616003954 PMC4822653 27048719

[bb12] Jelsch, C., Ejsmont, K. & Huder, L. (2014). *IUCrJ* **1**, 119–128. 10.1107/S2052252514003327 PMC4062089 25075328

[bb13] Kashiwagi, K., Pradhan, S., Haraguchi, T. & Akitsu, T. (2019). *J. Indian Chem. Soc.* **96**, 593–597.

[bb14] Keypour, H., Shooshtari, A., Rezaeivala, M., Valencia, L., Pérez-Lourido, P. & Khavasi, H. R. (2013). *Polyhedron* **50**, 104–110.

[bb15] Mastronarde, D. N. (2003). *Microsc. Microanal.* **9**, 1182–1183.

[bb16] McKinnon, J. J., Jayatilaka, D. & Spackman, M. A. (2007). *Chem. Commun.* pp. 3814–3816. 10.1039/b704980c 18217656

[bb17] Soni, K., Sharma, A., Akitsu, T. & Teotia, M. (2020). *J. Sci. Ind. Res.* **79**, 582–585.

[bb18] Spackman, M. A. & Jayatilaka, D. (2009). *CrystEngComm* **11**, 19–32.

[bb19] Tange, O. (2011). *;login: The USENIX Magazine* **36**, pp. 42–47.

[bb20] Watanabe, Y., Aritake, Y. & Akitsu, T. (2009). *Acta Cryst.* E**65**, m1640–m1641. 10.1107/S160053680904896X PMC2972068 21578655

[bb21] Westrip, S. P. (2010). *J. Appl. Cryst.* **43**, 920–925.

[bb22] Winter, G., Waterman, D. G., Parkhurst, J. M., Brewster, A. S., Gildea, R. J., Gerstel, M., Fuentes-Montero, L., Vollmar, M., Michels-Clark, T., Young, I. D., Sauter, N. K. & Evans, G. (2018). *Acta Cryst.* D**74**, 85–97. 10.1107/S2059798317017235 PMC5947772 29533234

[bb23] Yamane, S., Hiyoshi, Y., Tanaka, S., Ikenomoto, S., Numata, T., Takakura, K., Haraguchi, T., Palafox, M. A., Hara, M., Sugiyama, M. & Akitsu, T. (2017). *J. Chem. Chem. Eng.* **11**, 135–151.

