# Supplementary figures and images for: Crystal structure determination of an FeII azo aldehyde complex [Fe(C14H11N2O3)2(H2O)2] by MicroED
Source: Acta Crystallogr E Crystallogr Commun. 2026 May 12;82(Pt 6):544–50. doi: 10.1107/S2056989026004755 (PMC13239013; doi:10.1107/S2056989026004755)

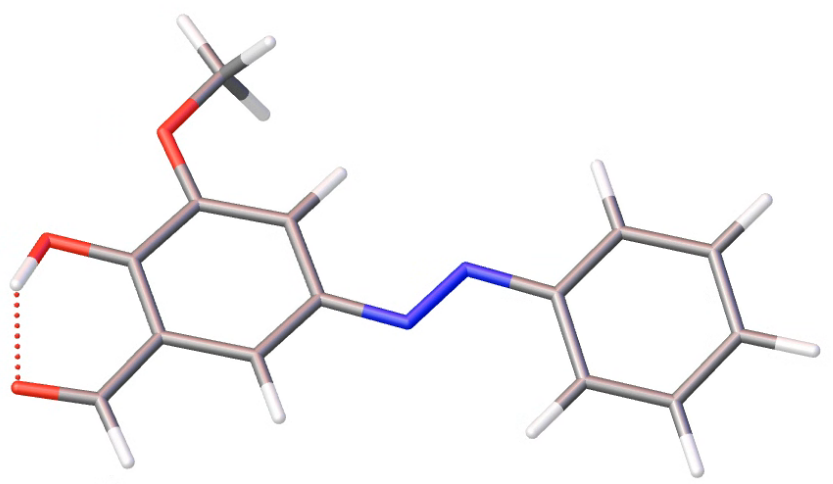

Supplement: Supplementary file 3 [file e-82-00544-sup3.tif]

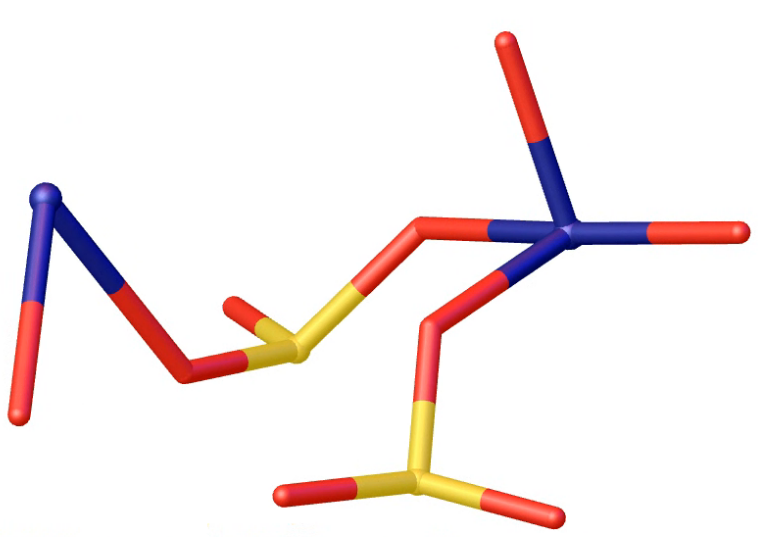

Supplement: Supplementary file 4 [file e-82-00544-sup4.tif]

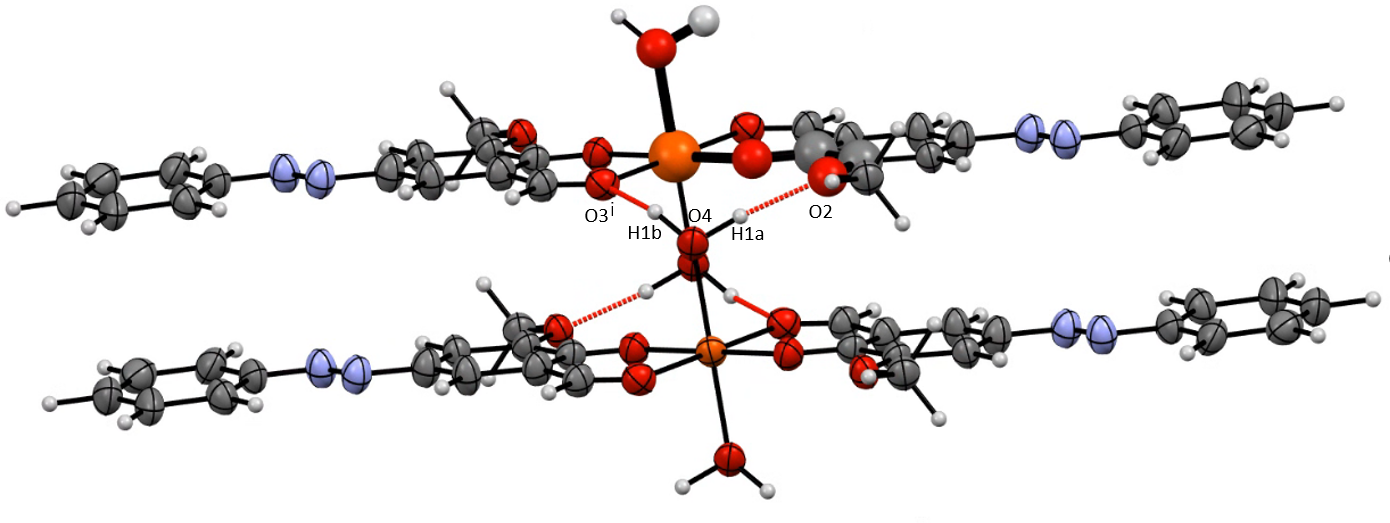

Supplement: Supplementary file 5 [file e-82-00544-sup5.tif]
